# Intervention Strategies to Elicit MVPA in Preschoolers during Outdoor Play

**DOI:** 10.3390/ijerph17020650

**Published:** 2020-01-19

**Authors:** Danielle D. Wadsworth, Jerraco L. Johnson, Alexandra V. Carroll, Melissa M. Pangelinan, Mary E. Rudisill, Julia Sassi

**Affiliations:** 1 Exercise Adherence and Obesity Prevention Laboratory, School of Kinesiology, Auburn University, 301 Wire Road, Auburn, AL 36849, USA; apv0004@auburn.edu; 2 Pediatric Movement Laboratory, School of Kinesiology, Auburn University, 301 Wire Road, Auburn, AL 36849, USA; johnson.8636@osu.edu (J.L.J.); mgp0020@auburn.edu (M.M.P.); rudisme@auburn.edu (M.E.R.); 3 Department of Human Sciences, The Ohio State University, Columbus, OH 43210, USA

**Keywords:** physical activity, fundamental motor skills, childcare

## Abstract

Approximately 50% of preschoolers do not meet physical activity recommendations and children who reside in low-income rural communities may be further at risk for higher levels of sedentary behavior. Outdoor play is essential for preschool children; however, literature is unclear as to which types of interventions elicit moderate-to-vigorous physical activity (MVPA) for all preschoolers. The aim of this study was to determine which type of intervention, physical activity or fundamental motor skill focus, elicits MVPA during outdoor play. Ninety-eight preschool children (M age = 4.48 years) from one Head Start center participated in an outdoor play intervention two days per week for 7 weeks. Classes were randomly assigned to one of four groups: fundamental motor skill focus (FMS), physical activity focus (PA), FMS and PA (FMS + PA), and control. An accelerometer worn on the hip measured MVPA. Results showed that age, sex and group assignment contributed to MVPA at the beginning of the intervention and age, sex, group assignment and MVPA during the beginning of the intervention contributed to MVPA at the end of the intervention. Overall, the FMS + PA group elicited MVPA from males and females of all ages. Interventions that combine both FMS and PA may reduce physical activity disparities in preschool children.

## 1. Introduction

Physical activity is essential for growth and development in young children and is associated with improved physical, behavioral, cognitive, and social outcomes [1,2]. In addition, physical activity is a contributing factor to decreasing the likelihood and rates of several chronic diseases among children [3]. Whereas, sedentary behavior is associated with cardiovascular disease and all-cause mortality in adults [3], and cardio-metabolic risk factors in children [4]. Current physical activity guidelines recommend preschool-aged children engage in three hours of light, moderate and vigorous physical activity throughout the day [3,5]. Although the benefits of physical activity are well documented, approximately 50% of preschool-aged children do not meet physical activity recommendations [5,6] and preschool children spend most of their time in sedentary activities [7].

Fundamental motor skills (FMS) and motor competence are related to physical activity participation in young children [8]. Previous research indicates that physical activity intensity increases during FMS practice, particularly in locomotor skills [9], suggesting that involvement in FMS may lead to increased physical activity levels and greater health benefits. In fact, a review by Figueroa and Anor [10] examined the relationship between motor skill competence and physical activity participation in preschoolers and found that a positive association between motor skill competence and physical activity participation has been consistently documented. Many of the studies included in this review utilized a cross sectional design, and thus, the strength of the relationship between motor skill competence and physical activity participation may be influenced by individual factors such as biological sex [11], age, and current level of physical activity [12]. However, a recent study examining motor competence across a large sample of children aged 3–6 years residing in the United States shows that approximately 77% of the sample examined were classified as delayed in FMS [13]. Thus, these children may be at an increased risk for lower levels of physical activity and potentially poorer health.

The low levels of physical activity coupled with delays in FMS calls for the development of intervention strategies aimed at children who demonstrate low levels of physical activity and FMS. Children from low-income families generally demonstrate a greater risk for lower physical activity, as well as long-term health disparities [14,15,16] and low and moderate socio-economic status schools provide fewer physical activity practices [17]. In addition, children who reside in rural, minority, and impoverished communities often demonstrate even lower levels of physical activity and higher levels of sedentary behavior than children who reside elsewhere because rural environments often lack the resources, infrastructure and supports to provide quality physical activity experiences [18]. Moreover, physical activity is influenced by demographic factors such as race and sex. African American and Hispanic children engage in less physical activity than non-Hispanic Caucasian children in the US [19]. Young females engage in less physical activity [20,21] and show further delays in FMS compared to males [13].

According to the National Center for Education Statistics, 40% of three-year-olds, 68% of four-year-olds, and 86% of five-year-olds were enrolled in preschool programs in 2017 [22], making preschool centers an ideal location to implement physical activity programs for young children. In order for preschool facilities to meet physical activity recommendations, they must provide opportunities for children to increase physical activity, as well as, engage in a variety of gross and fine movement activities [23]. Ward [24], Gordon [25], and their colleagues evaluated the evidence regarding the effectiveness of intervening in childcare centers and reported that environmental modifications (e.g., portable play equipment, floor markings) improve young children’s physical activity levels. The meta-analysis conducted by Gordon et al., [25] showed a small to moderate effect on preschoolers’ overall physical activity and a moderate effect on their level of moderate-to-vigorous physical activity (MVPA). These results support the notion that the early learning environment is an ideal setting to facilitate MVPA in preschoolers. A more recent review also reported a small but positive effect of childcare center interventions in increasing physical activity in young children [26]. Furthermore, these authors highlight the importance of outdoor play in increasing children’s physical activity levels.

Although evidence suggests that FMS and physical activity are interconnected, preschool interventions that examine outdoor play either focus on improving physical activity or FMS outcomes [10]. For example, a review by Gray et al., [27] investigated factors related to physical activity during outdoor play and found only one study out of 28 that examined motor skills. However, studies that have examined outcomes of play interventions in preschoolers have shown that interventions with a FMS focus can support physical activity [28]. For example, Wadsworth et al., [28] examined the effect of a year-long FMS focused intervention on physical activity levels in preschoolers and found that the intervention significantly increased physical activity compared to unstructured free play. What is not clear throughout the literature is if interventions aimed at improving FMS would confer a greater advantage over interventions that are specifically aimed at increasing physical activity levels directly. Furthermore, although both FMS [13] and physical activity [26] interventions have shown different effects for biological sex, no effort has been made to determine which type of intervention would be most beneficial for girls who consistently demonstrate lower physical activity and ball skills compared to their male counterparts. Finally, it is not clear how interventions should be tailored to benefit all children regardless of age, sex, and current level of physical activity, all of which have been postulated to influence physical activity outcomes [26]. This information is vital, as the relationship between motor skill competence and physical activity may be influenced by individual factors such as biological sex [11], age, and current level of physical activity [12] as well as environmental factors (e.g., setting, climate, intervention focus). Therefore, the aim of this study was to determine which type of intervention, physical activity or FMS focus, promoted greater levels of participation in physical activity for all preschool-aged children during outdoor play.

## 2. Materials and Methods

### 2.1. Participants

Ninety-eight (52 males, 46 females) preschool children aged 3 to 5 attending a local federally subsidized Head Start center participated in this study. Table 1 shows demographic characteristics of the sample. This particular center serves children and families who reside in low-income housing from surrounding rural communities. Preschool hours were from 8:00 am–12:30 pm and included breakfast (8:00–8:30), curriculum instruction including small/large group, centers and outdoor play time (8:30–11:30), lunch (11:30–12:00) and dismissal (12:30). A purposeful sampling was implemented as previous literature suggests that children from lower socio-economic status in rural communities are: (a) at risk for poor health, (b) more susceptible to developmental delays, and (c) do not meet recommended guidelines for physical activity [14,15,16,18].

Prior to data collection, this study was evaluated and approved by the Institutional Review Board and meets the latest Declaration of Helsinki (The protocol number for the approved IRB is: 06–262 EP 0701). Before gaining institutional approval, members of the research team met with the parental advisory council for the center to gain approval and input for the study. An informational letter and consent form were sent to the parents via weekly take-home folders. Out of a possible 106 children, 98 (92%) returned signed parental consent forms and assented to participate in the study. In terms of racial demographics, 92.9% of the sample was black, 5.1% Hispanic and 2% were white. Eight classrooms were randomly assigned to one of four high-autonomy groups: fundamental motor skill focus (FMS), physical activity focus (PA), fundamental motor skill and physical activity focus (FMS + PA) and a control group. As the relationship between motor skill competence and physical activity may be influenced by individual factors such as biological sex [11], age, and current level of physical activity [12] as well as environmental factors (e.g., setting, climate, intervention focus) [28,29], we hypothesized that preschool children randomly assigned to the PA and FMS + PS would exhibit higher levels of physical activity at the end of the intervention compared to preschool children in the FMS focused and control group. We also hypothesized that sex and MVPA in the beginning of the intervention would influence levels of physical activity at the end of the intervention.

### 2.2. Procedures

The physical activity program began in January and concluded in April, and was located on two outdoor playgrounds at the Head Start center. Physical activity data were gathered during outdoor play two times per week over nine weeks via accelerometry (Actigraph GT3X). Data were collected for a total of 14 sessions (7 weeks) with interruptions in data collection due to spring break (2 sessions) and a week of inclement weather (2 sessions). Each session lasted for 30 min and consisted of six to eight activity stations. Accelerometers were worn on the right hip and attached with an elastic belt by a researcher prior to the outdoor play and removed after outdoor play.

### 2.3. Intervention

The control group consisted of free play on the outdoor playground (no instruction). The physical play environment for the three experimental groups were high autonomy such that children were allowed to have complete autonomy over which stations they participated in, how long they stayed at these particular stations, as well as, who they played with (if anyone) while visiting these stations. A high autonomy climate was chosen because interventions for preschoolers utilizing high autonomy outdoor play is associated with higher levels of physical activity compared to free play, with children in high autonomy climates spending significantly more time in MVPA compared to free play (36% of time compared to 7%) [29]. High autonomy climates are also associated with improvements in fundamental motor skills for preschoolers [30] and considered developmentally appropriate for preschool children.

Each intervention play period consisted of six to eight stations that focused on FMS (hopping, running, galloping, jumping, throwing, catching, striking and dribbling) and physical activity. The stations for each experimental group were the same; however, the instructors manipulated the focus of the experimental condition. All equipment in all four conditions were the same and included a variety of balls, hoops, mats, goals, ground markings and ample space for movement.

For the FMS group, the instructors encouraged the children to learn motor skills by providing constant instruction and feedback regarding the technique and form of the skills at each station. At the beginning of each FMS session, a teacher would help the children warm-up by demonstrating FMS that corresponded to each station and the effective techniques for success from an FMS perspective. During the session, the teachers would provide feedback on FMS technique and proper FMS practice. For example, if a child in this group visited an overhand throwing station, the instructor would provide them with cues such as “reach your arm back” and “step with the opposite foot” to emphasize the importance of correct form in sport performance. If a child visited a station with running, the feedback was focused on proper form, including cues for “bent elbows, light on feet or toes and run tall”.

For the PA group, the instructors primarily encouraged the children to participate in as much physical activity as possible, regardless of whether or not they correctly performed the motor skill. At the beginning of the session, teachers helped the children warm-up by demonstrated possible physical activities at each station (running, hopping, dancing, etc.) and emphasized how much physical activity they could participate in. During the session, instructors constantly emphasized the importance of physical activity in health and sport performance. For example, at that same throwing station, the children were encouraged to throw the ball but the emphasis was on chasing the ball as fast as they could and retrieving it in order to exercise their heart and get physical activity. At a station that included galloping or running the emphasis was on doing as much as possible for as long as possible, versus proper execution of the skill. During this condition, the instructors never gave instruction on skill development.

For the FMS + PA group, the instructors equally encouraged the children to perform their skills correctly and encouraged children to get as much physical activity as possible. The warm-up consisted of both FMS instruction and physical activity and demonstrated both at each station. Instructors not only emphasized the importance of physical activity and exercise, but also addressed the importance of motor skill development. For example, a child at the throwing station was given cues such as “turn to the side before you throw” and also encouraged to retrieve the ball they threw. At a jumping station the child was encouraged to pull “both arms back and explode”, as well as, “to run through the station and try again.” The control group participated in unstructured free play on the opposite playground on the same days of the intervention. The equipment that was accessible to the control group was the same as the experimental conditions; however, no formal instructions were given.

Behavioral Fidelity. In order to confirm that each experimental condition conformed to the parameters defined, two reviewers viewed videotapes of eight 10-min lesson segments that were selected at random from each of the four conditions. These segments were evaluated on criteria from a modified climate fidelity checklist [31]. The checklist was modified to reflect how reinforcement and feedback were given in the climates in context to the four experimental conditions. For example, during the PA condition, was feedback and reinforcement given on physical activity or motor skill instruction? Fidelity assessments showed that overall the experimental groups met the criteria 99.3% of the video segments assessed. Specifically, the FMS group met the criteria 99.4%, the PA group met the criteria 97.8% and the FMS + PA and the control group met the criteria 100%. Agreement between the two reviewers was 96.4% across all four conditions.

### 2.4. Measurements

#### 2.4.1. Demographics and Anthropometrics

Participants’ date of birth, sex, and race were provided by parents on the parental consent form. Height and weight were measured in a private setting with children dressed in light clothing and shoes removed. Height was measured to the nearest 0.25 cm using a portable stadiometer. Weight was measured to the nearest 0.1 kg using a precision electronic scale.

#### 2.4.2. Physical Activity

Physical activity data were collected during the program on Tuesdays and Thursdays for a total of 7 weeks using Actigraph GT3X triaxial accelerometers (Mini-Mitter Co., Inc. Bend, OR, USA). Accelerometers were worn on the right hip and attached with an elastic belt by a researcher prior to the outdoor play and removed after outdoor play. ActiLife software was used to extract data, validate wear time, and compute physical activity levels. Each accelerometer was calibrated for each child based on height, weight, sex, and age according to manual guidelines. The accelerometers were programmed with 15-s epoch, which is recommended for preschool children [11] and MVPA was quantified by Butte [32] cut points. The cut-points correctly classified MVPA compared to direct calorimetry and double labeled water approximately 80% of the time. Previous studies report the reliability of the Actigraph accelerometer for preschoolers as 0.90–0.94 [33]. MVPA data were averaged across Tuesday and Thursday for each week.

### 2.5. Statistical Analysis

MATLAB (Version R2017a, Natick, MA, USA) was used for all statistical analyses with a level of significance *p* < 0.05. A stepwise linear regression (stepwiselm) was conducted to determine if there were differences in the percentage of time spent in MVPA during the first two weeks of the intervention. The stepwiselm function in MATLAB uses a sequential process in which the criterion for model terms to be added is based on the *p*-value of the *F*-statistic less than 0.05. At each step, the stepwiselm function checks for linear dependencies (i.e., if a term is redundant with another term). If a term is linearly dependent, the function removes the redundant term. The full model included the following terms: group (categorical-control, FMS, PA and FMS + PA), age (continuous), and sex (categorical-male, female). Interactions amongst these factors were also included in the statistical model. To determine differences in the percentage of time spent in MVPA during the last two weeks of the program and the factors that influenced these changes, a similar stepwise linear regression was run. The full model included the following terms: percentage of time spent in MVPA for the first two weeks of the program (weeks 1 and 2 averaged) as a covariate, group (control, FMS, FMS + PA, PA), age (continuous), and sex (male, female). Interactions amongst these factors were also included in the statistical model. Comparison of significant effects and interactions were decomposed using two sample or paired *t*-tests.

## 3. Results

### 3.1. Demographic Data

Table 1 shows the participants demographics. There was no difference in age between the four groups (*F*(3,94) = 0.26, *p >* 0.05) at the onset of the intervention.

### 3.2. MVPA

Table 2 presents the means and standard deviations for each group by sex for weeks 1 and 2 and weeks 6 and 7, as well as the results from the paired *t*-tests (with Cohen’s *d,* and *p*-values) conducted comparing weeks 1 and 2 and weeks 6 and 7 for each group by sex. The only participants that showed a significant increase in MVPA from weeks 1 and 2 to weeks 6 to 7 were the females in the control group (*t*(10) = −5.15, *d* = −1.56, *p* < 0.001). It is important to note that the interventions are underway during week 1 and 2, therefore, null results regarding changes from week 1 and 2 to weeks 6 and 7 for the intervention groups (FMS, FMS + PA, and PA) do not signify that the interventions were not successful.

**Figure 1:**
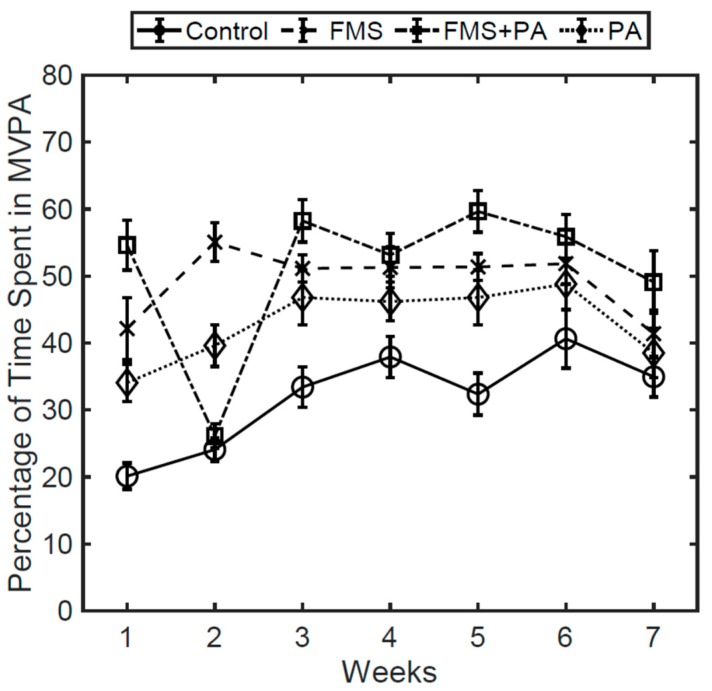
Percentage of time spent in MVPA by group across the intervention.

Figure 1 shows the percent of MVPA by group across the intervention. Indeed, although overall, there was a small increase in MVPA across the weeks of the intervention for the control group, the other groups maintained the same relative position across the weeks of the intervention (with the exception of the FMS group for week 2).

**Figure 2:**
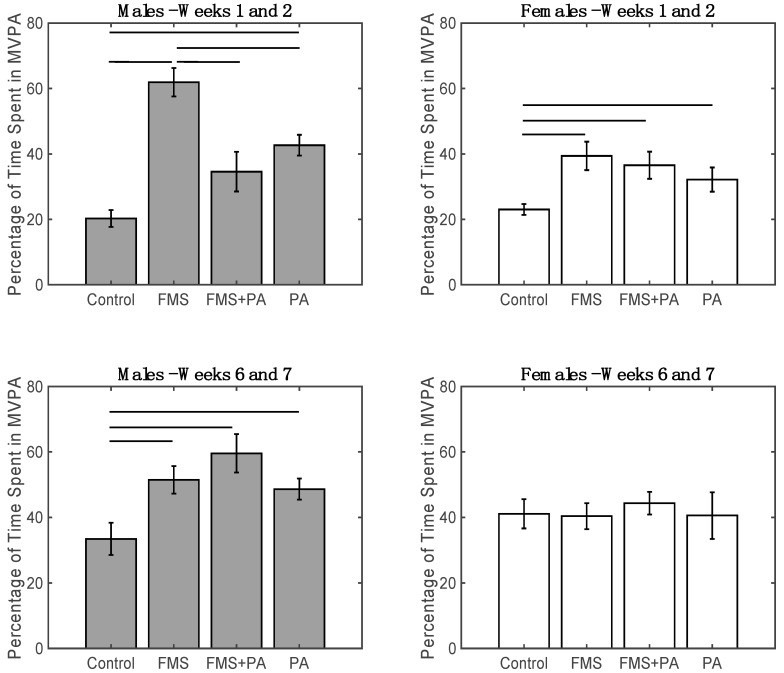
Top: Percentage of time spent in MVPA for weeks 1 and 2 by group (Control, FMS, FMS + PA, and PA) for males (Left) and females (Right). Bottom: percentage of time spent in MVPA for weeks 6 and 7 by group (Control, FMS, FMS + PA, and PA) by for males (Left) and females (Right). Means and standard errors are presented. Lines represent significant *t*-tests at *p* < 0.05.

The stepwise linear regression examining the factors that influence the percentage of time spent in MVPA for weeks 1 and 2 revealed main effect for Age (*F*(1,86) = 4.98, *p* < 0.05), where there was age-related increase in the percentage of time spent in MVPA for weeks 1 and 2. There were also main effects of Sex (*F*(1,86) = 6.52, *p* < 0.05), Group (*F*(3,86) = 18.85, *p* < 0.001), as well as a significant Sex x Group interaction (*F*(3,86) = 4.82, *p* < 0.01; Figure 2 Top). The full model accounted for 49.1% of variance (*F*(1,86) = 10.4, *p* < 0.001). Follow-up analysis of the Sex x Group interaction via two-sample *t*-tests revealed that the males in the Control group had significantly less time spent in MVPA for weeks 1 and 2 compared to the males in the FMS group (*t*(22) = −7.87, *p* < 0.001, *d* = −3.30) and males in the PA group (*t*(24) = −5.16, *p* < 0.001, *d* = −2.11). In addition, males in the FMS group spent significantly more time in MVPA for weeks 1 and 2 compared to the males in the FMS + PA group (*t*(22) = 3.57 *p* < 0.001, *d* = 1.44) and males in the PA group (*t*(26) = 3.64, *p* < 0.001, *d* = 1.37). For the females, those in the Control group had significantly less time spent in MVPA for weeks 1 and 2 compared to the females in the FMS group (*t*(22) = −3.53, *p* < 0.001, *d* = −1.44), FMS + PA group (*t*(21) = −3.01, *p* < 0.01, *d* = −1.24) and PA group (*t*(20) = −2.40, *p* < 0.05, *d* = −0.99). All other comparisons were not statistically significant (*p* > 0.05).

**Figure 3:**
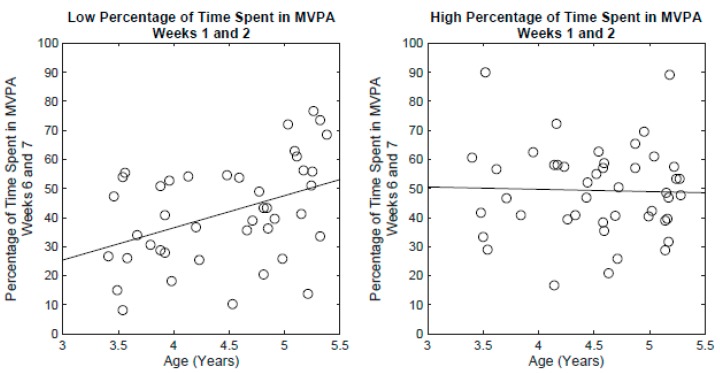
Left: Percentage of time spent in MVPA for weeks 6 and 7 by age for the participants with less than 35% time spent in MVPA for weeks 1 and 2. Each circle represents a participant. The line depicts the fitted regression. Right: Percentage of time spent in MVPA for weeks 6 and 7 by age for the participants with 35% or more time spent in MVPA for weeks 1 and 2. Each circle represents a participant. The line depicts the fitted regression.

The stepwise linear regression examining the factors that influence the percentage of time spent in MVPA for weeks 6 and 7 revealed main effect Age (*F*(1,76) = 6.61, *p* < 0.05), Group (*F*(3,76) = 3.07, *p* < 0.05), a Sex × Group interaction (*F*(3,76) = 2.77, *p* < 0.05; Figure 2 bottom), and an interaction between Age × percentage of time spent in MVPA for weeks 1 and 2. (*F*(1,76) = 6.27, *p* < 0.05; Figure 2). The full model accounted for 30.6% of variance (*F*(1,76) = 3.36, *p* < 0.01). Follow-up analysis of the Sex x Group interaction via two-sample *t*-tests revealed that after accounting for all other covariates, the males in the Control group spent significantly less time in MVPA for weeks 6 and 7 compared to the males in the FMS group (*t*(19) = −2.61, *p* < 0.05, *d* = −1.15), males in the FMS + PA group (*t*(19) = −3.08, *p* < 0.01, *d* = −1.38), and males in the PA group (*t*(21) = −2.51 *p* < 0.05, *d* = −1.05). In contrast, for the females, the difference in MVPA for weeks 6 and 7 between the controls and the other groups were not statistically significant (*p* > 0.05). For the follow-up analysis of the Age × percentage of time spent in MVPA for weeks 1 and 2 (Figure 3), the participants were split based on the median percentage of time spent in MVPA for weeks 1 and 2 (Low < 35%; High > = 35%). After accounting for all other covariates, a significant age-related increase in the percentage of time spent in MVPA for weeks 6 and 7 was observed for the Low group (*F*(1,40) = 8.20, *p <* 0.01, R-squared = 0.17), but not for the High group (*p >* 0.05).

## 4. Discussion

This study examined which type of intervention, physical activity or FMS focus, promoted greater levels of MVPA for all preschool-aged children during outdoor play. Our hypotheses were partially supported in that individual factors (i.e., sex and MVPA at the beginnig of the intervention) as well as environmental factors (i.e., group) influenced levels of MVPA at the end of the intervention.

The first major finding of this study was that intervention focus (i.e., environment) affected MVPA from the beginning to the end of the intervention. For example, although the girls in the control group showed an increase in MVPA from weeks one and two of the intervention to weeks six and seven, all other groups maintained the same (greater) level of MVPA across all weeks of the intervention. These results suggest that the intervention focus does indeed influence MVPA at the outset of the program (see below for additional influential factors for MVPA during weeks six and seven).

The second major finding was an age-related increase in MVPA at weeks one and two of the intervention, where older children demonstrated higher levels of MVPA than younger children, which is aligned with recent findings in which sedentary time decreased and MVPA increased as children aged [34]. This is in contrast to previous findings reported, which demonstrated that younger children (three-year-olds) were more active than older children (four- to five-year-olds) [35]. Pate et al., [35] hypothesized that older children may have more time in structured activities that require more sedentary time; however, this was not the case for children in our study. MVPA differences across groups were observed during the first two weeks of the study for males and females in the different groups. Overall, males showed higher levels of MVPA than girls, which is consistent with previous research [21,34,35]. However, males in the control group had lower levels of MVPA compared to males in the FMS + PA group. Furthermore, males in the FMS group showed higher levels of MVPA compared to males in the FMS + PA intervention, as well as, males in the PA intervention. These results suggest that the FMS intervention, in particular, is more effective in benefitting males MVPA levels, which is consistent with recent findings showing males having higher levels of MVPA in structured activities than females [36]. Females in the control group showed lower levels of MVPA compared to all of the other intervention groups. This effect may be due to females preferring social and structured forms of play versus free play [37] and impacted by level of fundamental motor skill.

The third major finding was that in addition to age, sex, and intervention type, children’s MVPA level during the early part of the intervention also had an influenced MVPA in weeks six and seven. Inconsistent improvements in MVPA were observed for weeks six and seven across the groups. Males and females in the control group increased MVPA from weeks one and two (as stated above); however, the males in the control group still exhibited significantly lower MVPA compared to the intervention groups. This finding has been observed in other intervention studies, where children in control groups demonstrate gains over time, but still show less improvement in MVPA compared to the intervention groups [38,39]. The group that showed the largest difference (compared to the controls) were the males in the FMS + PA group. With that being said, there was no significant differences between the intervention groups for either the males or females. These results suggest that the FMS + PA group influenced MVPA in both males and females, regardless of their age or MVPA at the onset of the intervention. There was no significant difference in MVPA in weeks six and seven across females in the control group and intervention groups, suggesting that the control group caught up to the intervention groups. Finally, an age-related increase in MVPA was seen in children that began the intervention with low levels of MVPA. Although this extends the findings from weeks one and two, this finding suggests that over six weeks, the older children may increase MVPA to a greater extent than young children regardless of which intervention group they were in.

The present study focused on the types of interventions that would elicit changes in MVPA during outdoor play. Although young children may achieve bouts of MVPA throughout the day, young children may be able to sustain higher levels physical activity for an extended period of time during outdoor play. The time window examined was only 30 min, which was the time allotted for outdoor play at the Head Start center. Although children in the control group increased MPVA over the seven-week intervention, on average males in this group achieved about 10 min of MVPA and females achieved about 12 min of MVPA. In contrast, the males in the FMS + PA were able to achieve over 17 min of MVPA and the females in this group achieved over 13 min of MVPA. These results suggest that young children engaging in non-structured outdoor play may have fallen short of meeting the 15 min per hour recommendation, whereas the males in the FMS + PA group met the recommendation for that hour. This has several implications and practical applications for incorporating outdoor play in early childhood settings. First, when designing outdoor play activities for preschoolers’ activities, which reinforce both FMS and PA are equally beneficial for males and females across age. Second, males exhibit higher levels of physical activity in outdoor play climates that focus on FMS.

This study is not without limitations. First, the duration of the study was only seven weeks long. However, we did collect data across the entire seven weeks of the study. Future studies should determine if factors such as initial physical activity impact longer interventions. Second, we were unable to determine the impact of different environments (e.g., different types of preschool programs), as this only examined one preschool center. Finally, we purposely sampled low-income minority children from rural environments, as these children are the most at risk for low levels of physical activity; however, this selective recruitment strategy decreases the generalization of the study.

## 5. Conclusions

This seven-week intervention was effective at determining which type of interventions elicit changes in MVPA in preschool-aged children during outdoor play. We found that age, sex, intervention approach, and MVPA during the beginning of the intervention all influence preschoolers’ MVPA. However, physical play environments that emphasize motor skill development and physical activity participation (i.e., FMS + PA) may elicit higher levels of MVPA and decrease physical activity disparities for all preschoolers. These findings provide new insights to address the knowledge gap regarding how to elicit MVPA in outdoor play programs to benefit all preschoolers. Future studies should determine if FMS + PA interventions are effective at increasing physical activity levels throughout the day for preschool children.

## Figures and Tables

**Table 1:** Demographics [means and standard deviations (SD)].

Group	N (Females)	Mean Age (SD)	Mean Height in Inches (SD)	Mean Weight in Lbs. (SD)
Control	23 (12)	4.53 (0.67)	41.87 (2.97)	42.79 (9.45)
Fundamental motor skills (FMS)	25 (12)	4.45 (0.62)	43.00 (2.47)	42.55 (5.75)
FMS + physical activity (PA)	25 (12)	4.40 (0.64)	42.52 (3.29)	41.46 (7.89)
PA	25 (10)	4.54 (0.59)	42.73 (1.73)	44.15 (8.10)

**Table 2:** Percentage of time spent in moderate to vigorous physical activity (MVPA) (means and standard deviations) for each group by sex for weeks 1 and 2 and weeks 6 and 7. *t*-statistics, Cohen’s *d,* and *p*-values are presented for the paired *t*-tests comparing weeks 1 and 2 with weeks 6 and 7 by group and sex.

Group	Sex	Mean % MVPA Weeks 1 and 2 (SD)	Mean % MVPA Weeks 6 and 7 (SD)	*t*-Statistic	Cohen’s *D*	*p*-Value
Control	Male	20.27 (8.62)	33.45 (16.23)	*t*(8) = −2.13	*d* = −1.01	*p* = 0.07
	Female	23.00 (5.77)	41.08 (15.38)	*t*(10) = −5.15	*d* = −1.56	*p* < 0.001
FMS	Male	61.94 (15.62)	51.48 (15.19)	*t*(11) = 2.04	*d* = 0.68	*p* = 0.07
	Female	39.39 (15.03)	40.40 (13.60)	*t*(11) = −0.25	*d* = −0.07	*p* = 0.81
FMS + PA	Male	34.58 (21.87)	59.58 (21.14)	*t*(9) = −2.05	*d* = −1.16	*p* = 0.07
	Female	36.55 (14.38)	44.36 (12.05)	*t*(9) = −1.80	*d* = −0.59	*p* = 0.11
PA	Male	42.68 (12.34)	48.62 (12.50)	*t*(13) = −1.17	*d* = −0.48	*p* = 0.26
	Female	32.17 (11.69)	40.56 (22.40)	*t*(8) = −1.32	*d* = −0.47	*p* = 0.22
